# Dynamics of HIV-1 Quasispecies during Antiviral Treatment Dissected Using Ultra-Deep Pyrosequencing

**DOI:** 10.1371/journal.pone.0011345

**Published:** 2010-07-07

**Authors:** Charlotte Hedskog, Mattias Mild, Johanna Jernberg, Ellen Sherwood, Göran Bratt, Thomas Leitner, Joakim Lundeberg, Björn Andersson, Jan Albert

**Affiliations:** 1 Department of Microbiology, Tumor and Cell Biology, Karolinska Institutet, Stockholm, Sweden; 2 Department of Virology, Swedish Institute for Infectious Disease Control, Stockholm, Sweden; 3 Science for Life Laboratory Stockholm, Solna, Sweden; 4 Venhälsan, Stockholm South General Hospital, Stockholm, Sweden; 5 Department of Clinical Science and Education, Karolinska Institutet, Stockholm, Sweden; 6 Theoretical Biology and Biophysics, Los Alamos National Laboratory, Los Alamos, New Mexico, United States of America; 7 Division of Gene Technology, School of Biotechnology, Royal Institute of Technology, AlbaNova University Center, Stockholm, Sweden; 8 Department of Cell and Molecular Biology, Karolinska Institutet, Stockholm, Sweden; University of California San Francisco, United States of America

## Abstract

**Background:**

Ultra-deep pyrosequencing (UDPS) allows identification of rare HIV-1 variants and minority drug resistance mutations, which are not detectable by standard sequencing.

**Principal Findings:**

Here, UDPS was used to analyze the dynamics of HIV-1 genetic variation in reverse transcriptase (RT) (amino acids 180–220) in six individuals consecutively sampled before, during and after failing 3TC and AZT containing antiretroviral treatment. Optimized UDPS protocols and bioinformatic software were developed to generate, clean and analyze the data. The data cleaning strategy reduced the error rate of UDPS to an average of 0.05%, which is lower than previously reported. Consequently, the cut-off for detection of resistance mutations was very low. A median of 16,016 (range 2,406–35,401) sequence reads were obtained per sample, which allowed detection and quantification of minority resistance mutations at amino acid position 181, 184, 188, 190, 210, 215 and 219 in RT. In four of five pre-treatment samples low levels (0.07–0.09%) of the M184I mutation were observed. Other resistance mutations, except T215A and T215I were below the detection limit. During treatment failure, M184V replaced M184I and dominated the population in combination with T215Y, while wild-type variants were rarely detected. Resistant virus disappeared rapidly after treatment interruption and was undetectable as early as after 3 months. In most patients, drug resistant variants were replaced by wild-type variants identical to those present before treatment, suggesting rebound from latent reservoirs.

**Conclusions:**

With this highly sensitive UDPS protocol preexisting drug resistance was infrequently observed; only M184I, T215A and T215I were detected at very low levels. Similarly, drug resistant variants in plasma quickly decreased to undetectable levels after treatment interruption. The study gives important insights into the dynamics of the HIV-1 quasispecies and is of relevance for future research and clinical use of the UDPS technology.

## Introduction

Human immunodeficiency virus type 1 (HIV-1) displays very high genetic variability, which is the primary obstacle for development of an effective HIV vaccine and the reason for the emergence of resistance during antiretroviral therapy (ART). Within an HIV-1 infected individual, selective pressures, such as the host immune response and ART, influence the evolution of the virus. This leads to the formation of a diverse pool of closely related virus variants called a quasispecies [1], [2]. The genetic diversity is caused by the error-prone reverse transcriptase (RT), which generates an average of 3.4×10^−5^ mutations per site and generation [3], [4], the high virion production rate and the short generation time [5], [6], [7], [8]. Finally, recombination events that occur during reverse transcription also contribute to genetic variability [9], [10]. Consequently, point mutations, including those associated with drug resistance, are spontaneously generated many times every day even in patients who never have received ART [5]. Even though recent data indicate that minority drug resistance variants may be associated with reduced treatment efficacy in treatment-naïve individuals [11], [12], little is still known about variation in the relative abundance of preexisting resistance mutations and if such variation has clinical significance.

Drug resistance does not generally develop in patients who are adherent to modern combination antiretroviral treatment (cART), but may develop very quickly during suboptimal treatment. Primary resistance mutations are often associated with a fitness cost, and therefore resistant virus variants are usually replaced by wild-type variants if cART is interrupted. Studies have suggested that these rebounding wild-type variants originate either from wild-type virus that had been archived in latently infected cells before start of therapy [13] or from continued evolution that leads to reversion of resistance mutations [14], [15].

Newly developed high-throughput sequencing technologies have revolutionized genetic research. One such technology is massive parallel pyrosequencing [16]. One application of this technology is ultra-deep pyrosequencing (UDPS), which allows identification of rare genetic variants and minority drug resistance mutations, which are not detectable by standard genotypic sequencing techniques [12], [17], [18], [19].

The aim of this study was to use UDPS to investigate the *in vivo* dynamics of HIV quasispecies in longitudinally collected plasma samples from six individuals who started treatment before the cART era. We analyzed a region of *pol* corresponding to amino acids 180–220 in the RT. This region includes the following important and well-defined drug resistance mutations to nucleoside RT inhibitors (NRTIs) and non-nucleoside RT inhibitors (NNRTIs): L210W, T215Y/F and K219Q/E associated with resistance to zidovudine (AZT) and stavudine (d4T); M184I/V associated with resistance to lamivudine (3TC) and emtricitabine (FTC); and Y181C/I/V, Y188C/L/H and G190S/A associated with resistance to nevirapine (NVP), efavirenz (EFV) and etravirin (ETR) [20]. We also studied so called T215 reversion mutations (T215A/C/D/E/G/H/I/L/N/S/V) [21]. As the name indicates these mutations are usually seen in patients who have failed and later interrupted therapy with zidovudine (AZT) or stavudine (d4T), which leads to “reversion” of the resistance mutations T215Y and T215F, but nothing precludes that they may be present as minority variants before therapy.

The sensitivity for detection of rare variants is primarily determined by the number of virus templates that can be successfully extracted and amplified from plasma samples and by the error rate of PCR and UDPS [4]. Here, we have developed optimized protocols to maximize HIV template input and new bioinformatic software to clean the sequence data from PCR and sequencing errors, which allowed us to detect genuine virus variants that constituted as little as 0.05% of the HIV-1 quasispecies. This has to our knowledge not been achieved before in studies of HIV-1 resistance. Interestingly, we found that even with our highly sensitive UDPS methods, preexisting drug resistance was infrequently observed. Thus, only M184I, T215A and T215I were found at very low levels. During treatment failure, wild-type variants were below the detections limit in all except one patient. Finally, after treatment interruption drug resistant variants in plasma decreased to undetectable levels as early as after three month, which may be important for clinical management of patients with previous treatment failure since our results suggests that drug resistant variants is difficult in plasma even with this sensitive technology. Moreover, our findings give important insights into the dynamics of the HIV-1 quasispecies and are of relevance for future research and clinical use of the UDPS technology.

## Materials and Methods

### Ethical statement

Ethics application was approved by Regional Ethical Review board in Stockholm, Sweden (Dnr 52/2008-77). Patients participating in this study gave written informed consent according to the Declaration of Helsinki.

### Plasma samples

**Figure 1:**
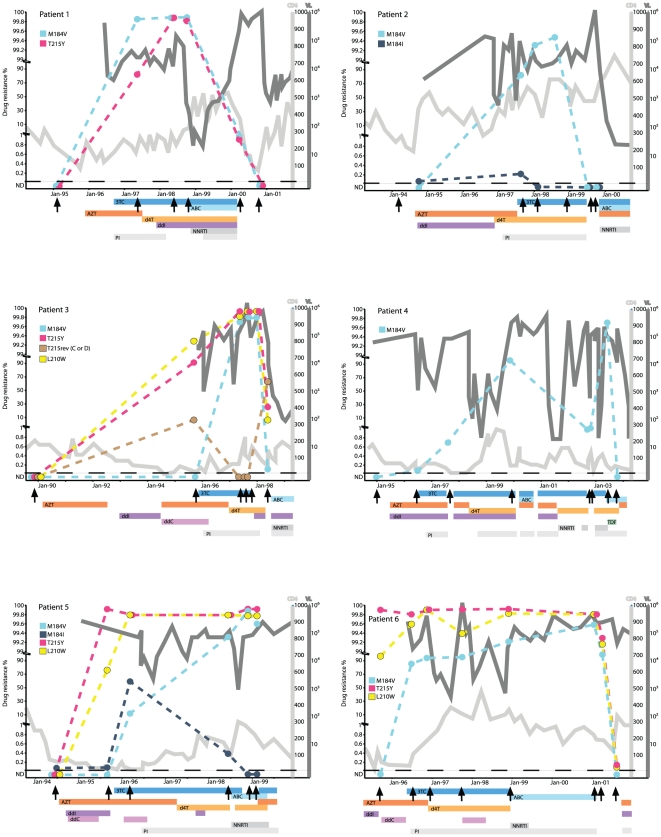
Frequency of resistance drug resistance mutations M184V, T215Y, L210W and T215C/D before, during and after treatment. Treatment history is indicated by bars below each patient's graph; AZT; zidovudine, 3TC; lamivudine, d4T; stavudine, ddI; didanosine, ABC; abacavir, ddC; zalcitabine, TDF; tenofovir, NNRTI; non-nucleoside reverse transcriptase inhibitors, PI; protease inhibitors. Arrows indicate time for sampling. The dashed horizontal lines indicate the detection limit in each patient. ND: not detectible.

A total of 40 plasma samples from six HIV-1 subtype-B-infected individuals were included in the study. Patients participating in this study gave written informed consent according to the Declaration of Helsinki. From each patient, longitudinally collected plasma samples that had been stored at −70°C or −20°C, were selected based on the patients' treatment history and plasma viral load (ranging from 17,900–1,600,000 HIV-1 RNA copies/mL). Information about the patients and the samples is summarized in Table 1 and Fig. 1. All patients had experienced virological treatment failure and all patients, except one, had later undergone treatment interruption. The treatment histories of the patients differed, but all started therapy before the cART era and all had received regimens that contained 3TC, AZT and d4T. Five of six patients were sampled before any treatment was initiated. All patients were sampled two to four times during treatment, i.e. the first possible sample after treatment initiation and additional samples obtained during therapy failure. Finally, five patients were sampled during treatment interruption.

**Table 1:** Characteristics of the patient samples and basic information on UDPS.

Patient	Sample	Treatment a	Sampling b	RNA copies/ml c	Vol. (µL)^d^	Yield (%)^e^	No. of templ. f	Readsbefore j	Reads after k
1	1	None	1995-02-23	274,000	500	2.4	3,300	18,541	15,000
	2	3TC,AZT,PI	1997-04-09	41,300	1000	24	10,000	20,785	15,418
	3	3TC,d4T,ddI	1998-04-02	55,500	1000	7.2	4,000	21,682	17,257
	4	3TC,d4T,ddI	1998-09-09	58,700	1000	12	7,000	20,553	16,958
	5	None	2000-02-28	210,000	1000	48	100,000	23,376	18,037
	6	None	2000-09-21	966,000	500	100	570,000	21,811	18,141
2	1	None	1994-01-10	284,000	500	2.8	4,000	16,847	13,414
	2	3TC,d4T,PI	1997-08-13	73,600	1000	9.5	7,000	22,113	19,291
	3	3TC,d4T,PI	1998-01-27	17,900	1000	48	8,600	18,693	16,649
	4	3TC,d4T,PI	1998-12-07	67,700	1000	8.9	6,000	17,949	15,890
	5	None	1999-09-02	1,600,000	200	3.1	10,000	17,672	16,142
	6	None	1999-09-29	371,000	500	2.4	4400	13,928	11,107
3	1	None	1989-10-16	20,000	1000	45	9,000	21,714	18,767
	2	AZT, ddC	1995-10-31	19,400	1000	12	2,300	3,837	2,406
	3	3TC,d4T,PI	1997-05-27	74,800	1000	19	14,000	22,191	17,693
	4	3TC,d4T,PI	1997-08-18	248,600	800	45	90,000	35,033	28,423
	5	3TC,d4T,PI	1997-10-02	2,171,200	500	13	144,000	23,420	18,202
	6	None	1998-05-04	385,000	1000	47	180,000	11,495	9,355
4	1	None	1994-11-15	54,400	1000	20	10,800	11,898	9,781
	2	AZT, ddI	1996-06-03	112,000	1000	14	16,000	22,477	15,866
	3	None	1997-07-02	83,100	1000	41	34,000	35,954	25,253
	4	3TC,d4T,ddI,PI	1999-12-14	331,000	750	44	108,000	23,429	17,511
	5	None	2002-08-16	160,000	1000	9.6	15,300	11,489	10,098
	6	None	2002-10-13	356,000	800	59	166,600	10,874	9,375
	7	ABC,d4T,TDF,PI	2003-02-24	234,000	800	25	47,600	9,405	7,641
	8	ABC,d4T,PI	2003-09-15	68,200	1000	40	27,000	11,909	10,288
5	1	None	1994-06-07	40,000	1000	9.0	3600	22,974	19,104
	2	AZT, ddI	1995-08-08	46,400	1000	5.0	2,300	23,625	18,299
	3	3TC,AZT,ddC	1996-03-26	245,000	800	16	31,000	10,548	7,770
	4	3TC,d4T,PI	1998-05-07	117,000	800	16	15,000	24,166	16,965
	5	ABC,d4T,NNRTI,PI	1998-11-13	87,200	1000	18	16,000	22,449	15,575
	6	ABC,d4T,NNRTI,PI	1999-01-26	235,000	800	1.3	2,400	20,918	15,157
6	1	AZT	1995-08-17	542,000	500	46	126,000	20,979	15,422
	2	3TC,AZT,PI	1996-06-19	224,700	800	16	29,000	8,290	5,906
	3	3TC,d4T,PI	1996-12-05	375,600	500	43	81,000	12,146	8,783
	4	3TC,d4T,PI	1997-10-21	1,054,800	200	19	40,000	38,541	29,582
	5	ABC,NNRTI,PI	1998-12-02	21,600	1000	17	3,600	41,940	35,401
	6	ABC,PI	2001-01-04	349,000	500	18	32,000	11,004	8,182
	7	None	2001-02-15	200,000	600	1.9	2,300	24,727	20,082
	8	None	2001-07-02	250,000	600	1.8	2,700	29,233	25,038

Footnotes

a 3TC; lamivudine, ABC; abacavir, AZT; zidovudine, d4T; stavudine, ddI; didanosine, ddC; zalcitabin, TDF; tenofovir, EFV; efavirenz, NNRTI; non-nucleoside reverse transcriptase inhibitor; PI; protease inhibitor.

b Time point for sampling.

c In plasma.

d Plasma volume used for extraction.

e RNA recovery after extraction/cDNA synthesis efficiency.

f cDNA templates subjected to UDPS.

j UDPS reads before cleaning.

k UDPS reads after cleaning.

### RNA extraction, cDNA synthesis and PCR amplification

Considerable effort was invested in evaluating and comparing different approaches for RNA extraction, cDNA synthesis and PCR amplification, and substantial differences between methods were observed (data not shown). The aim of the protocol was to maximize the number of plasma HIV RNA molecules that were extracted, reverse transcribed, PCR amplified and finally subjected to UDPS. The final optimized protocol is presented below and a detailed workflow is outlined in Fig. S1. HIV RNA was extracted and purified with the RNeasy Lipid Tissue Mini Kit (Qiagen, Hilden, Germany) using the QIAvac 24 vacuum minifold protocol (Qiagen, Hilden, Germany) and the RNA was eluted in 40 µl of RNase free water. The amount of plasma used for extraction was adjusted to the viral load (200 µL–1000 µL). The extracted viral RNA (40 µL) was divided into five aliquots of 8 µl and reverse transcribed with Thermoscript (Invitrogen, Carlsbad, California, US) using the gene specific primer JA272 (see below) according to the manufacturer's instructions. The five 20 µl cDNA aliquots from each sample were pooled into a total volume of 100 µl cDNA before PCR with outer primers. The HIV-1 cDNA copy number, i.e. actual number of viral templates subjected to UDPS, was quantified for each sample using an in-house limiting dilution PCR method adapted from Brinchmann *et al*. [22].

The cDNA was used to amplify 261 nucleotides spanning amino acid positions 163 to 223 (position 3070 to 3209 in HXB2, GenBank accession number K03455) of the reverse transcriptase (RT) region of the pol gene with a nested PCR approach using the Fast Start High Fidelity System (Roche, Penzberg, Germany). The total 100 µl volume of cDNA was divided into parallel outer PCR reactions each containing 5 µl of cDNA. Next, the products from the outer PCR were pooled and 2.5 µl was used in two inner PCR reactions. Both PCRs were carried out as follows: one initial denaturation step of 95°C for 2 min followed by 30 cycles of denaturation for 20 s at 94°C, annealing for 20 s at 50°C, and extension for 90 s and a final 6-min extension at 72°C. The primers used were, outer sense primer JA269, outer antisense JA 272, inner sense JA329 and inner antisense JA331 (for details on primers see Table S1). The inner sense and antisense primers were linked to UDPS adapters A and B, respectively. To distinguish each sample in the multiplexed UDPS, eight unique sequence tags were inserted between the adaptor and the gene specific primer (for details on the tag sequences see Table S2).

### Ultra deep pyrosequencing (UDPS)

Before UDPS the PCR amplicons were purified using the GE PCR purification kit (GE health care, Pollards Wood, United Kingdom) and the DNA concentration and purity was determined using Nanodrop (Thermo Fisher Scientific, Waltham, US). In addition, the Agilent 2100 bioanalyzer (Agilent Life Science, Santa Clara, California, US) was used to verify the quality and length of the amplicons. After quality controls, PCR amplicons from eight samples were pooled in equimolar concentrations and sequenced in both forward and reverse direction on the 454 Life Science platform (GS-FLX, Roche Applied Science) according to the manufacturer's instructions.

### Sanger sequencing

To facilitate the UDPS data cleaning processes and to verify the sample authenticity the *pol* gene of all samples was also subjected to direct population Sanger sequencing (ABI Prism 3100) using Big Dye terminator cycle sequencing kit according to recommendations by the manufacturer (Applied Biosystems, Foster City, California, US). To identify possible contamination between specimens the population sequences were used to reconstruct neighbor-joining phylogenetic trees with the MEGA 4.0 software using the maximum composite likelihood model with gamma distributed rates across sites (α = 0.5) [23]. Statistical support for internal branches in the tree was obtained by 1000 bootstrap replicates. The sequences showed a patient-specific clustering with high bootstrap support (>90%), which argues against the occurrence of contamination and sample mix-up (data not shown).

### Control experiments

#### PCR and UDPS errors

To measure the accuracy of our UDPS protocol, the SG3Δenv plasmid was diluted to 1 copy, amplified using the optimized protocols described above and subjected to UDPS. The entire procedure from sample preparation to UDPS was repeated three times. The sequence of the plasmid clone was determined by Sanger sequencing and any difference from the Sanger sequence in the UDPS analysis was assumed to be a PCR or UDPS error. Based on these data we estimated the average frequency sequencing errors in the analyzed fragment as well as the frequency of sequencing errors at each nucleotide position. We calculated statistically derived cut-off values for detection of all possible mutations at each position.

#### *In vitro* recombination

The frequency of *in vitro* recombination during the PCR was evaluated by mixing two clones. The plasmid clones were generated from patient samples using TOPO TA cloning kit (Invitrogen, Carlsbad, California, US). Two plasmid clones, that contained 14 informative sites disbursed over the amplicon, were mixed in equal proportions and diluted to 10,000 templates and 100,000 templates before PCR amplification and UDPS.

### Data cleaning strategy

New bioinformatic software was written to manage, clean and analyze the UDPS data (Jernberg *et al.*, manuscript in preparation). The software was inspired by Tsibris *et al*. [24] who kindly made their code available to us before publication. Since eight samples were analyzed simultaneously by UDPS in each physical field of the Picotiter plate, reads from each individual sample were first identified using the sample-specific sequence tags in the primers (see Table S2). Next, the data was cleaned by a set of scripts that discarded; 1) all reads with <80% similarity to the corresponding Sanger sequence, 2) reads containing ambiguous bases (Ns), 3) reads that did not cover the region of interest (amino acids 180–220 in RT, position 3087 to 3206 in HxB2, GenBank accession number K03455). Remaining reads were imported into the GS amplicon software (Roche, Penzberg, Germany) and aligned. The alignment was extracted and the amount of data was compressed by scripts that identified unique sequence variants in forward and reverse direction and counted the number of reads per variant. The tally for each variant was retained with the sequence name for further analyses. The alignment was cut to the region of interest (amino acid 180–220) and gaps were removed. Since UDPS errors are known to be concentrated to homopolymeric regions, reads with out-of frame insertions or deletions were removed. Finally, the alignments were manually inspected and any remaining variants with frameshifts or stop codons were removed.

After editing, the tallies for the forward and reverse sequence of each variant were compared and the abundance of the variant was set to the sum of the forward and reverse tallies. However, if frequency of the forward and reverse reads differ by more than a factor of 10 we made the assumption that a systematic error had occurred during 454 sequencing and adjusted the frequency to the lower of the two estimates. Finally, the variant was discarded from further analyses if the variant was absent in either forward or reverse direction.

### Drug resistance analyses

Individual cut-off values were calculated for all drug resistance mutation positions using the clone data obtained from three different UPDS runs, which we refer to as 1, 2 and 3. The nucleotide sequences were translated into amino acid sequences. Three error rates (1, 2 and 3) for all positions were calculated using all reads in every position. The error rates were calculated by estimating the number of mismatches between all the UDPS reads generated from the SG3Δenv plasmid and the corresponding Sanger sequence.

The error rates (1, 2 and 3) were combined to one average error rate and a 95% confidence interval was calculated. This was estimated for every drug resistance mutation (Jernberg *et al*. manuscript in preparation). Based on these error rates and the number of reads from the clone data and the number of reads in each patient sample we calculated individual cut-off values (p-value <0.05) for all resistance mutations in all samples using a Chi-square test with correction for continuity.

### Variant analysis

Variants were classified as high-confidence variants or as probable sequencing artifacts in the following way. The Needleman-Wunsch algorithm was used to construct pairwise alignment between the Sanger sequence of the SG3Δenv plasmid and UDPS clonal reads. The errors per nucleotide from all pairwise comparisons were added together and divided by the number of reads. Each of the three data sets was analyzed separately and an overall average and cut-off values were calculated in the same way as for the drug resistance analyses. Variants with prevalence higher than the cut-off values were classified as high-confidence variants and were retained for further analyses and variants below the cut-off values were discarded. The genetic distance of each variant from the most prevalent variant in the first sample of the patient was calculated by computing pair-wise distance in MEGA 4.0 using the Tamura-Nei model with gamma distributed rates across sites (α = 0.5) [23]. The total nucleotide diversity between all high-confidence variants in each patients sample was determined by computing the average pair-wise distance weighted according to the prevalence of each such variant.

## Results

### Sample preparation and UDPS data cleaning

In this study we have used the UDPS technology to dissect the HIV-1 quasispecies evolution in longitudinally collected plasma samples from six HIV-1 infected individuals. The pre-UDPS protocols, i.e. the RNA extraction, cDNA synthesis and PCR, were carefully optimized for high recovery. The number of recovered cDNA molecules was quantified by limiting dilution PCR and compared to the HIV-1 RNA levels of the original plasma samples. These analyses showed that the number of cDNA molecules subjected to UDPS ranged from 2,300 to 570,000 and that the RNA extraction and cDNA synthesis methods had a combined efficiency that ranged from 1.3% to 100% (Table 1). The low efficiency of preparation for some samples could possibly be explained by sample storage conditions since most samples had been stored at −70°C or −20°C for long time and sometimes also had been repeatedly freeze-thawed. From the UDPS we obtained a total of 800,615 reads with a median of 20,949 (range 3,837 to 41,490) reads from each sample, which agreed well with the 25,000 reads per sample that we had aimed at. The UDPS data were cleaned to remove reads with PCR and UDPS artifacts while retaining as many high-confidence sequences as possible (see Materials and Methods). During this process a median of 20% (range 9%–37%) of the reads were discarded from each sample so that a median of 16,016 (range 2,406–35,401) reads remained from each sample. Information about treatment, plasma viral levels, number of viral templates recovered and number of reads before and after data cleaning are shown in Table 1.

### Validation of UDPS

New technologies such as UDPS need to be validated. Therefore, we performed several control experiments. The results from these experiments were used to investigate UDPS sequencing errors, determine *in vitro* recombination rates, calculate the sequencing depth and compute cut-off values for detection of resistance mutations and minority sequence variants.

#### Estimation of UDPS error rate and cut-off values for detection of minority resistance mutations

To measure the accuracy of the UDPS, we sequenced the SG3Δenv plasmid clone in three separate UDPS runs, which generated a total of 45,679 sequence reads (after data cleaning). From these data we estimated that the average error rate of UDPS after data cleaning was less than 0.05% errors per nucleotide compared to 0.54% before data cleaning. Thus, our cleaning strategy decreased the error rate approximately 10-fold. The error rate was not uniform across sites (Jernberg *et al*, manuscript in preparation). For this reason we calculated the UDPS error rates for each nucleotide position associated with drug resistance and used the upper limit of the 95% confidence interval as cut-off value for detection of mutations. The cut-off values for the resistance mutations relevant for this study are summarized in Table 2. As illustrated in Table 2, the possibility to detect minority resistance mutations depends not only on the UDPS error rate, but also on the sequence depth. Thus, cut-off values for detecting resistance mutations were individually calculated for every sample using a Chi-square test with correction for continuity.

**Table 2:** Estimated cut-off values for detection of selected drug resistance mutations.

Mutation	Upper limit (n = 2406)^a^	Median (n = 16016)^b^	Lower limit (n = 35401)^c^
Y181C	0.17%	0.07%	0.06%
Y181I	0.04%	0.02%	0.01%
Y181V	0.04%	0.02%	0.01%
M184V	0.29%	0.17%	0.16%
M184I	0.17%	0.07%	0.06%
Y188C	0.25%	0.15%	0.13%
Y188H	0.25%	0.14%	0.12%
Y188L	0.04%	0.02%	0.01%
G190A	0.04%	0.02%	0.01%
G190S	0.04%	0.02%	0.01%
L210W	0.04%	0.02%	0.01%
T215Y	0.04%	0.02%	0.01%
T215F	0.04%	0.02%	0.01%
T215I	0.17%	0.07%	0.06%
T215N	0.04%	0.02%	0.01%
T215S	0.04%	0.02%	0.01%
T215A	0.08%	0.02%	0.02%
K219Q	0.04%	0.02%	0.01%
K219E	0.04%	0.02%	0.01%

Footnotes

a The upper limit cut-off value represent the 95% confidence interval for a sample with 2406 reads, which was the lowest number of reads analyzed from a patient sample in this study.

b The median cut-off value represent the 95% confidence interval for a sample with 16016 reads, which was the median number of reads analyzed in the patients samples in this study.

c The lower limit cut-off value represent the 95% confidence interval for a sample with 35401 reads, which was the highest number of reads analyzed from a patient sample in this study.

#### Low frequency of *in vitro* recombination

The frequency of *in vitro* recombination during the PCR was evaluated by UDPS analysis of 100, 000 and 10,000 templates from a mixture of two clones, which differed at 14 nucleotide positions. The overall proportion of recombinant sequences prior to data cleaning was 0.76% and 0.27%, respectively. After data cleaning, no recombinant variants were detected in the 10,000 template mixture while two recombinant variants were detected in the 100,000 template mixture at proportions of 0.22% and 0.18% of the total number of sequences, respectively. Thus, PCR recombination was rare and is unlikely to have influenced our results.

### Prevalence of drug resistance mutations before, during and after failing treatment

#### Significant preexistence of the M184I, T215A and T215I mutations

Pre-treatment samples were available for five of the six patients. The M184I resistance mutation was detected in the virus populations from four of five patients (patients 1, 2, 3, and 5) at levels that ranged from 0.07% to 0.09% (Table 3). The M184I mutation confers high-level of resistance (about 1000-fold) to 3TC [25] and during treatment failure it is known to appear transiently before being replaced by M184V [26], [27]. We also investigated the levels of preexisting T215 reversion mutations (T215A/C/D/E/G/H/I/L/N/S/V) and found that four of five patients had preexisting levels of T215A and/or T215I that ranged from 0.05% to 0.11% (Table 3), whereas we did not detect any of the other 215 reversion mutations. Our results show that T215A and T215I not only evolve following treatment interruption in patients with failing therapy, but also can exist as minority variants prior to any therapy.

**Table 3:** Prevalence of drug resistance mutations in pre-treatment plasma samples.

Patient	Proportion of resistance mutation % a																		
	Y181C	Y181I	Y181V	M184V	M184I	Y188C	Y188H	Y188L	G190A	G190S	L210W	T215Y	T215F	215I^b^	T215N^b^	T215S^b^	215A^b^	K219Q	K219E
1	0.02	0	0	0.03	**0.09**	0.05	0.01	0	0	0	0	0.001	0	**0.1**	0.01	0	0.01	0.02	0.01
2	0.02	0	0	0.05	**0.08**	0.02	0.04	0	0	0	0	0	0	0.07	0.02	0	0.02	0	0
3	0.02	0	0	0.11	**0.07**	0.03	0.08	0	0	0	0.01	0.01	0	**0.08**	0	0.01	**0.05**	0	0.01
4	0.02	0	0	0.09	0.02	0.05	0.11	0	0	0	0	0	0	**0.11**	0	0	0.01	0	0.04
5	0.03	0	0	0.12	**0.07**	0.03	0.05	0	0	0	0	0	0	0.03	0.03	0.01	**0.05**	0	0.04

Footnotes

a Numbers in bold are above the statistical cut-off values.

b Other T215 reversion mutations, T215C/D/E/G/H/L/V, were not detected.

#### No detectable pre-existence of the M184V, T215Y/F and NNRTI resistance mutations

None of the five individuals had significant levels of Y181C/I/V, M184V, Y188C/L/H, G190S/A, L210W, T215Y/F and K219E before treatment (Table 3). Thus, we did not detect significant pre-treatment levels of the three important NRTI resistance mutations (M184V, T215Y and T215F) nor the three important NNRTI mutations (Y181C/I/V, Y188C/L/H and G190S/A). However, it should be noted that M184V was observed, but the levels of this mutation were not above the statistically derived cut-off value, because the error rate was comparably high for this mutation (median cut-off value 0.17%) (Table 2).

#### Transient increase of M184I during treatment failure

From patients 2 and 5, we had samples taken three and four months after the start of 3TC containing therapy, respectively. At this time, the M184I mutation had increased from 0.08% to 0.27% in patient 2 and from 0.08% to 63% in patient 5 (Fig. 1). However, the M184I mutation was completely replaced by M184V after 8 months of 3TC treatment in patient 2 and 3 years in patient 5. In the three remaining patients no detectable levels of M184I was observed in the first available sample after start of 3TC therapy, which was obtained between 9 month to 2 years (patients 1, 3 and 4) after start of therapy. Instead, in these patients the M184V mutation dominated and was found in 99.5–99.9% of the virus population (Table 4).

**Table 4:** Highest levels of drug resistance detected during treatment.

Patient	Mutation %		
	M184V	L210W	T215Y
1	99.9	-	99.9
2	99.5	-	-
3	99.8	99.9	99.9
4	99.9	0.117	-
5	99.8	99.8	99.9
6	99.6	99.9	99.9

#### During treatment failure almost 100% of the virus population displays resistance mutations

Drug resistance mutations evolved during suboptimal treatment in all six patients. All patients developed the M184V mutation, four patients developed T215Y and three developed L210W. The M184V mutation increased in prevalence during failing 3TC-containing regimen in all patients and finally constituted between 99.5% and 99.9% of the viral quasispecies (Fig. 1 and Table 4). In two of the four patients (patients 1 and 3) the T215Y mutation also increased gradually from 78 and 90.3% after approximately 16 months of AZT-containing treatment to 99.9% 12 months and 19 months later, respectively. In the remaining two patients who developed the T215Y mutation (patients 5 and 6), no gradual increase was observed and a prevalence of 99.9% was seen after 5 to 13 months of treatment (Fig. 1 and Table 4).

Surprisingly, AZT/d4T-associated mutations did not develop in patients 2 and 4 despite failing AZT/d4T containing regimen, which could indicate that the adherence to AZT/d4T treatment was too low to drive development of resistance [28]. Taken together, drug resistance developed quickly in all patients and increased gradually until almost the entire quasispecies was resistant.

#### Almost complete disappearance of resistance mutations from plasma virus during treatment interruption

Five of the six patients were sampled during treatment interruption. In patient 6 the level of resistance was still high 2 weeks after treatment interruption when M184V and T215Y was found in 98.5% and 99.3% of the quasispecies, respectively (Table 5, Fig. 1). However, after prolonged treatment interruption the prevalence of the resistance mutations rapidly decreased and after five month the M184V mutation was undetectable and the T215Y present at 0.10% (Table 5). In patient 1 and 4 low frequencies of M184V were found 1 and 3 months after treatment interruption, respectively, representing 2.3% and 3.9% (Table 5). In contrast, patient 2 and 4 had undetectable levels of M184V already 3 and 6 months after 3TC interruption, respectively (Table 5). Thus, the M184V resistance mutations decreased quickly after treatment interruption in all five patients. However, the rate of decay of M184V varied and after 3–8 months the mutation was not detectable in virus from plasma.

**Table 5:** Prevalence of drug resistance mutations during treatment interruption.

Patient	Sample	Stop of treatment		Mutations %^c^								
		3TC/ABC^a^	AZT/d4T^b^	M184V	M184I	L210W	T215Y	T215C	T215D	T215A	T215G	T215I
1	5	1 mo	1 mo	**2.32**	0.04	0	**1.03**	0	0	0	0	0.05
	6	8 mo	8 mo	0.01	0.06	0	0	0	0	0.01	0	**0.08**
2	5	3 mo	-	0.07	0.06	**0.02**	0	0	0	0	0	0
	6	4 mo	-	0.07	0.06	0	0	0	0	0.01	0	0.01
3	6	6 mo	3 mo	0.14	**0.1**	**9.68**	**27.6**	**62.3**	**6.04**	0.01	0.01	0
4	3	1 mo	-	**0.71**	0.06	**0.02**	**0.02**	0	0	**0.02**	0	**0.07**
	5	1 mo	-	**1.07**	**0.11**	**0.04**	0	0	0	**0.03**	0	0.07
	6	3 mo	-	**3.88**	**0.1**	**0.12**	0	0	0	**0.06**	0	0.04
6	7	0.5 mo	2 yr 4 mo	**98.5**	0.01	**99.2**	**99.3**	0.02	0	0.01	0	0
	8	5 mo	2 yr 9 mo	0.04	0.04	**0.04**	**0.1**	0	0	0.02	0	**0.6**

Footnotes

a 3TC, lamivudine; ABC, abacavir.

b AZT, zidovudine; d4T, stavudine.

c Numbers in bold are above the statistical cut-off values.

### Dynamics of HIV-1 variants

As described above our control experiments using mixtures of HIV clones showed that *in vitro* recombination was rare with our experimental protocol. This allowed us to track individual HIV variants over time. Thus, we determined the number of variants in each sample to investigate the population dynamics of HIV-1 in our six patients. The number of variants ranged from 221 to 1729 in the six patients, but most variants were only represented by a small number of reads and their frequency did not exceed the statistically derived cut-off values for detection of high-confidence variants (see Materials and Methods). The number of high-confidence variants ranged from 17 to 76 (Table 6).

**Table 6:** Number of significantly detected viral variants throughout the study period for each patient.

Patient	Sample 1	Sample 2	Sample 3	Sample 4	Sample 5	Sample 6	Sample 7	Sample 8
1	*57* a	**17** b	**38**	**33**	17^c^	30	-	-
2	*72*	**23**	**44**	**32**	22	21	-	-
3	*59*	**76**	**30**	**30**	**25**	49	-	-
4	*73*	**76**	**70**	**43**	**35**	43	21	32
5	*65*	**53**	**40**	**44**	**24**	**46**	-	-
6	**63**	**42**	**38**	**31**	**35**	**44**	54	31

Footnotes

*^a^*Variants in italic are sampled before any treatment is initiated.

b Variants in bold are sampled during treatment failure.

c Variants in normal text are sampled during treatment interruption.

#### Decreasing number of genetic variants during development and reversion of resistance

In all patients the number of genetic variants decreased over time and there were significantly fewer variants present in the last sample as compared to the first sample from each patient (p = 0.028, Wilcoxon matched pairs test) (Table 6). This suggests that the viral populations had undergone genetic bottlenecks during the development and reversion of resistance. We also analyzed changes in genetic diversity over time and did not find any changes that clearly could be related to changes in treatment (Table S3). However, it should be pointed out that the study was not designed to analyze changes in diversity.

#### High degree of linkage between drug resistance mutations during treatment failure

**Figure 2:**
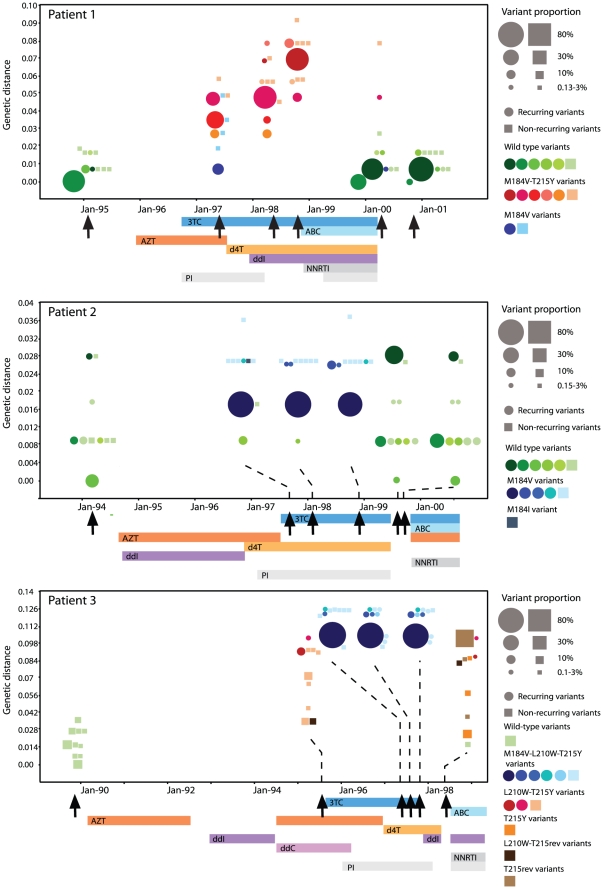
HIV-1 variant dynamics before, during and after treatment. For each patient the 10 most common variants in each time point are illustrated as circles (if recurring) or as cubes (if not recurring). The genetic distance of the variants in nucleotide changes/site (from the most frequent variant at the first time-point) is plotted over time. The frequency of the variants is proportional to the area of the circles and cubes. Treatment history is indicated by bars below each patient's graph; AZT; zidovudine, 3TC; lamivudine, d4T; stavudine, ddI; didanosine, ABC; abacavir, ddC; zalcitabine, TDF; tenofovir, NNRTI; non-nucleoside reverse transcriptase inhibitors, PI; protease inhibitors. Arrows indicate time for sampling. The genotype of the variants is color-coded, thus each combination of drug resistance mutations have a specific color (see guide to the right unique for each patient). There are at maximum six shades of each color enable means to follow specific variants over time. Thus, the most common variant receives the first shade and so on. The last shade is used for the remaining variants and for the non-recurring variants.

**Figure 3:**
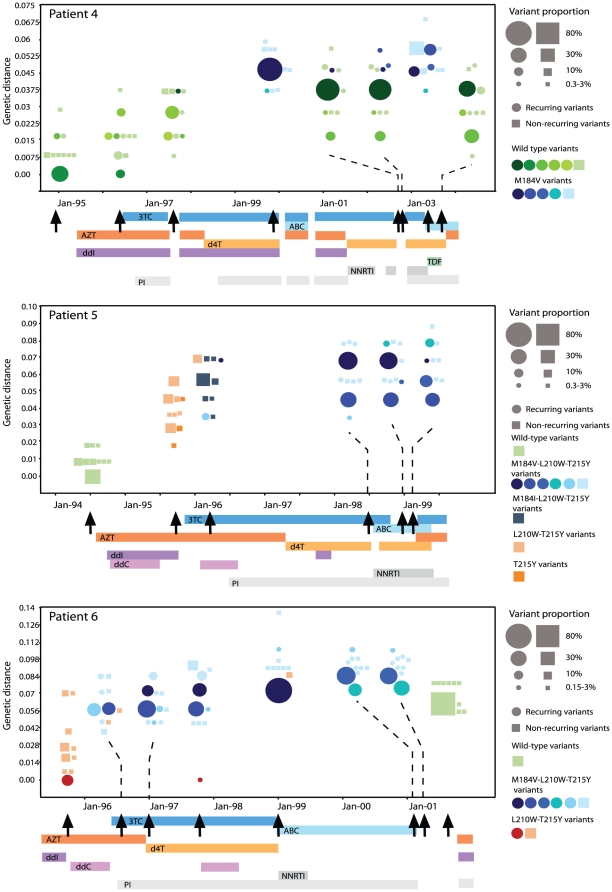
HIV-1 variant dynamics before, during and after treatment. For each patient the 10 most common variants in each time point are illustrated as circles (if recurring) or as cubes (if not recurring). The genetic distance of the variants in nucleotide changes/site (from the most frequent variant at the first time-point) is plotted over time. The frequency of the variants is proportional to the area of the circles and cubes. Treatment history is indicated by bars below each patient's graph; AZT; zidovudine, 3TC; lamivudine, d4T; stavudine, ddI; didanosine, ABC; abacavir, ddC; zalcitabine, TDF; tenofovir, NNRTI; non-nucleoside reverse transcriptase inhibitors, PI; protease inhibitors. Arrows indicate time for sampling. The genotype of the variants is color-coded, thus each combination of drug resistance mutations have a specific color (see guide to the right unique for each patient). There are at maximum six shades of each color enable means to follow specific variants over time. Thus, the most common variant receives the first shade and so on. The last shade is used for the remaining variants and for the non-recurring variants.

In Figure 2 and Figure 3 the 10 most common variants in each time-point and their genetic distance from the most common variant in the first sample is plotted for each patient. Patient 1 started on AZT monotherapy in the end of 1995 and had 3TC added eight months later (Fig. 2, patient 1). Nine months after 3TC introduction variants that only had the M184V mutation co-existed with variants with linked M184V and T215Y mutations. However, during prolonged 3TC containing treatment the M184V variants were completely replaced by the M184V-T215Y variants, suggesting that the M184V-T215Y variants were more fit during selection pressure from 3TC, d4T and ddI. It is interesting to note that several different M184V-T215Y variants co-existed, suggesting that they did not all arise through recombination but by convergent selection on these sites. Similar patterns were observed in all patients in whom more than one drug resistance mutation emerged (patients 2 and 3 in Fig. 2, patient 5 and 6 in Fig. 3).

During maximum levels of resistance no wild-type variants were detected in plasma from five of the six patients. However, in patient 2 wild-type variants were detected in the first two samples during treatment (Fig. 2). Since this patient did not develop the T215Y/F mutations despite a failing AZT containing regimen, we cannot exclude problems with adherence [28]. Thus, wild-type variants were rarely detected in the replicating plasma virus population during treatment failure, even with our highly sensitive assay.

#### Treatment interruption resulted in reappearance of archived wild-type variants or reversion of resistance through continued evolution

In four of the six patients (patients 1, 2, 3 and 4) we were able to analyze the origin of the drug-sensitive variants that re-appeared during treatment interruption. For patient 1, 2 and 4 the reappearing drug-sensitive variants were identical to wild-type variants that were present before therapy was initiated (Fig. 2 and Fig. 3). In patients 1 and 4, none of drug sensitive variants were detected during treatment, suggesting that the rebounding drug-sensitive variants originated from archived virus in latent reservoirs. For patient 2, two different drug-sensitive variants were detected during treatment. None of these existed before treatment, whereas one of them represented 7.7% of the quasispecies after treatment interruption. In patient 3, a completely different pattern was observed and none of the variants detected before treatment were observed after treatment interruption. Instead, the drug-sensitive variants that appeared after 3 and 6 months after 3TC and d4T treatment interruption, respectively, had reversion mutations at position 215 (Fig. 2). None of these “reversion” variants were detected before or during therapy, suggesting that they evolved from resistant variants rather than originated from archived virus in latent reservoirs. Finally, none of the 10 most common variants during treatment interruption had the M184V mutation, which was present in multiple variants during treatment failure. This suggests that this mutation had independently reverted to wild-type in several different drug resistant variants. For patient 5 and 6 we were not able to draw any conclusions on the origin of non-resistant virus during treatment interruption since samples before treatment (patient 6) or after treatment (patient 5) were not available.

## Discussion

In this study, we have used the UDPS technology to study the evolution of drug resistance and to detect minority virus variants in HIV-1 *pol* from six longitudinally followed patients. We optimized all pre-UDPS protocols and developed new data cleaning strategies. This allowed us to identify minority resistance mutations and viral variants that constituted as little as 0.05% of the viral quasispecies, enabling detailed studies of the viral dynamics during ART. We found no or very low levels of drug resistance before treatment, but during treatment failure resistant viruses effectively out-competed wild-type variants and constituted almost 100% of the viral population. When treatment was interrupted drug resistant viruses disappeared rapidly and were undetectable in plasma as early as after 3 months.

The sensitivity of UPDS depends on the number of viral templates that can be successfully extracted and amplified from a plasma sample [12], [29], the error rate of PCR and UDPS and efficiency of cleaning the UDPS data from such errors. In previous UDPS studies of HIV resistance, the sensitivity usually has been limited by template numbers because relatively small sample volumes were used and the actual number of templates subjected to UDPS was low or not quantified [18], [19], [30], [31]. Furthermore, the sequence depth has been relatively low, i.e. approximately 500–3600 reads per base in these studies of HIV-1 resistance [12], [18], [19], [30], [31]. Therefore, the lower limit of detection of minor resistance mutations was estimated to be 0.5–3.0% in the previous studies [12], [18], [19], [30], [31]. Here, we have used optimized pre-UDPS protocols (i.e. RNA extraction, cDNA and PCR) and quantified the number of HIV cDNA templates subjected to UDPS, which ranged from 2,300 to 570,000. Furthermore, we have sequenced considerably deeper (median 20,949 reads, range 3,837–41,940) than earlier investigators. For these reasons the sensitivity of our UDPS was primarily limited by errors introduced during PCR and UDPS. Thus, it was a challenge to distinguish rare, but genuine, variants from sequencing artifacts. By analyzing the frequency and distribution of sequencing errors in experiments on plasmid clones we were able to develop bioinformatic software to clean data from sequencing artifacts and to determine statistical cut-off values for detection of high-confidence minority resistance mutations and genetic variants. The error rate across sites was 0.05% (95% confidence, upper limit) after data cleaning, which is lower than the previously reported 0.1% to 1%[16], [17], [18], [19], [24], [32]. Consequently, our cut-off values for detection of high-confidence resistance mutations and viral variants are considerably lower than in previous studies. As expected, the error rate was not uniform across sites. For this reason we estimated the UDPS error rate for each drug resistance position (Jernberg *et al*, manuscript in preparation). These cut-off values were also dependent on the number of reads, but resistance mutations that represented on average 0.05% (range 0.014–0.29%) and viral variants that represented on average 0.11% (range 0.09–0.21%) were high-confidence, i.e. exceeded our statistically derived cut-off values. Thus, by using optimized pre-UDPS protocols and effective data cleaning strategies, we have been able to increase the sensitivity for detection of genuine virus variants so that variants within the HIV-1 quasispecies that are as rare as 1 in 1000 can be reliably detected. This has to our knowledge not been achieved before for HIV resistance studies. However, comparable detection limit was reported in one study of the HIV-1 envelope gene [24]. For some samples the number of sequence reads exceeded the number of viral templates, which means that some templates were resampled. Such resampling affects the sequencing depth, since it is not possible to sequence deeper than the number of input templates. However, we quantified the number of templates and the lowest number of templates was 2,300, resulting in a theoretical depth of 0.04% (1/2300), but for most samples the template input number was higher.

Mutations associated with drug resistance are expected to occur naturally within the HIV quasispecies, even if a patient has never received ART [5]. By simple calculations, using a reverse transcriptase error rate of 3.4×10^−5^ mutations per site and generation [3], [4] and viral production rate of 10^10^ [7] it can be estimated that all nucleotides in the HIV-1 genome on average mutate about 10^5^ times per day in an HIV-1 infected individual. However, it is not known at what frequency these mutations are present and if they in some situations may be of clinical relevance. In this study we found significant levels of M184I (4 of 5 patients), T215I and/or T215A (4 of 5 patients) ranging from 0.02%–0.12% in plasma samples obtained before treatment was initiated. In contrast, we did not find any significant pre-existence of the major drug resistance mutations M184V, Y181C, Y188C or T215Y/F. The presence of M184I, T215I and T215A in treatment naïve patients is somewhat expected since these drug resistance mutations only differ by one nucleotide from wild-type. For the same reason we would have expected to find M184V, Y181C and Y188C, but not T215Y/F since the latter are double mutants compared to wild-type. One explanation for the absence of significant levels of M184V, Y181C and Y188C could be that the cut-off values were higher at these positions (e.g. 0.15% for M184V compared to 0.07% for M184I) or that these mutations are associated with a higher fitness cost. However, Johnson *et al* [33] developed sensitive real-time PCRs and estimated the absolute assay sensitivities on a clone as well as the natural occurrence of several resistance mutations, including M184V, Y181C, T215Y and T215F, in 138 treatment naïve patients with samples collected before the ART era. They found evidence of natural occurrence of all four resistance mutations at low levels and the cut-off values for detection of high-confidence minority resistance mutations (transmitted or acquired) were determined to be 0.5%, 1.0%, 1.0% and 0.7%, respectively. Since the detection limits of our UDPS technology are below these cut off values, (0.15%, 0.07%, 0.02% and 0.02%, respectively) it is surprising that we did not find any of these mutations in our pre-treatment samples.

It is interesting to note that we found significant levels of M184I, but not M184V, before treatment in 4 of 5 patients. This agrees with early data from 3TC mono-therapy studies where it was shown that M184I usually occurs transiently before being replaced by M184V, which is more fit in the presence of 3TC [26], [34]. Several possible explanations have been proposed for the transient occurrence of M184I. Our data indicate that the primary reason may be a higher pre-treatment level, which in turn may be due to one or several of the proposed underlying mechanisms. It has been proposed that this initial appearance of M184I is due to the balance between mutational bias of RT and selective pressure. For HIV, G-to-A mutations are more common than other mutations. Thus, there is a higher production rate of M184I than M184V since the wild-type methionine is coded ATG and the resistance mutations to isoleucine and valine are coded ATA and GTG, respectively [34], [35]. In addition, *in vitro* studies have shown that the mutation rate from wild-type to M184I is more than four times higher than that to M184V, while the enzymatic efficiency of a RT with M184I is approximately 50% lower than that of a RT with M184V [36]. Therefore, the bias for G-to-A mutations of HIV-1 works in favor for M184I, while the selective pressure for enzymatic efficiency selects for M184V.

Our analyses of HIV-1 variants showed that different wild-type variants co-existed before initiation of therapy. Following start of therapy, virus variants with several different combinations of resistance mutations evolved and co-existed (Fig. 2 and Fig. 3). However, during prolonged treatment failure the number of viral variants decreased, suggesting genetic bottle-necking. This was accompanied by a gradual increase in the prevalence of variants with specific linked drug resistance mutations (in particular variants with M184V+T215Y and M184V+L210W+T215Y) (Table 4; Fig. 2 and Fig. 3) and wild-type variants were only detected in one patient during therapy. This finding indicates that wild-type variants had very low fitness during therapy and that very little wild-type virus was produced from viral reservoirs in latently infected cells, such as memory CD4+ T-lymphocytes. However, it is likely that wild-type variants still were present in plasma at levels below our detection limit of 0.05% because it is well known that there is residual viremia during long-term successful ART, that these viral variants often are drug-sensitive [37], [38], [39] and that this residual viremia is due to virus release from stable reservoirs of infection [40]. In contrast to our results, Allers *et al*. reported significant levels (0.6 to 30%) of lamivudine-sensitive variants in viral population from patients with failing 3TC-containing therapy [41]. However, these patients received dual-therapy with 3TC and AZT, while our patients generally received three or more drugs.

Since many drug resistance mutations reduce replication fitness [42], drug-sensitive viruses rapidly evolve after complete treatment interruption [13], [43]. However, the kinetics and detailed dynamics of this process are largely unknown. In this study we have shown that drug resistant variants decreased to undetectable levels a few months after complete treatment interruption. This indicates that these resistant variants have very low fitness in the absence of therapy. The complete out-growth of drug-sensitive variants within a few months differs markedly from the findings in patients with transmitted drug resistance, where drug resistance may persist for many years [44], [45], [46]. Our findings might be of clinical relevance since we show that drug resistant variants may be very difficult to detect in patients with previous treatment failure even with highly sensitive UDPS technology. At some variance with our data, Le *et al*. found low abundance mutations associated with AZT/d4T resistance 2 to 7 years after treatment with these drugs had been stopped. However, in contrast to our patients, the patients enrolled in their study continued therapy with other antiviral drugs. Thus, additional studies are needed to investigate the dynamics of drug resistant variants after treatment interruption. Since drug resistant variants can become established in long-term reservoirs [44], [47], it would be interesting to analyze different cell compartments in addition to plasma.

In conclusion, we have developed optimized UDPS protocols that have decreased the UDPS error rate and thereby increased the sensitivity for detection of minority HIV-1 resistance mutations and viral variants. With this technology we were able to identify and quantify variants that represented as little as 0.05% of the HIV-1 quasispecies. We have shown that the levels of preexisting drug resistance in plasma samples from treatment naïve patients is very low and that several important drug resistance mutations (M184V, Y181C, Y188C and T215Y/F) were not detectable in pre-treatment samples, indicating that the natural occurrence of these mutations are below our detection limit. Furthermore, there was almost 100% replacement of wild-type and drug-resistant variants during treatment failure and treatment interruption, respectively. Thus, our study shows that UDPS can be used to gain new insights in HIV evolution and resistance and is relevant for the possible future clinical use of this technology.

## Supporting Information

Figure S1 Workflow over the optimized protocol.
(3.00 MB TIF)

Table S1 Primers used for PCR amplification.
(0.03 MB DOC)

Table S2 Tag sequences.
(0.03 MB DOC)

Table S3 Genetic diversity of the 10 most common variants at each sampling time point.
(0.03 MB DOC)
