# Supplementary figures and images for: Dynamics of HIV-1 Quasispecies during Antiviral Treatment Dissected Using Ultra-Deep Pyrosequencing
Source: PLoS One. 2010 Jul 7;5(7):e11345. doi: 10.1371/journal.pone.0011345 (PMC2898805; doi:10.1371/journal.pone.0011345)

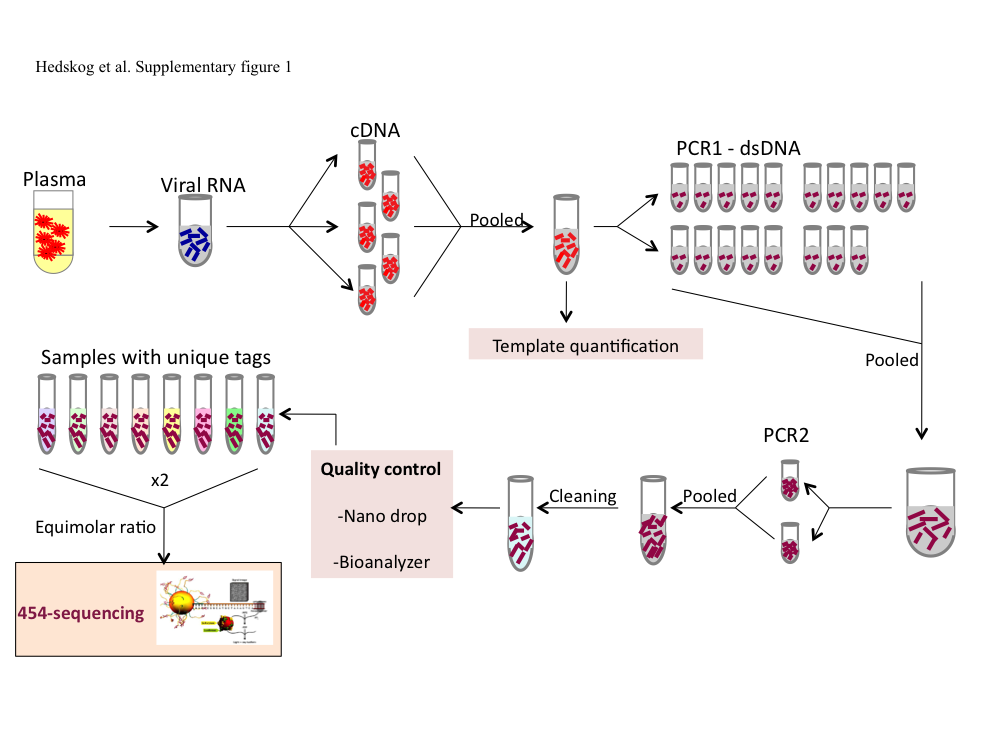

Supplement: Figure S1 — Workflow over the optimized protocol. (3.00 MB TIF) [file pone.0011345.s001.tif]
