# Supplementary material for: Dynamics of HIV-1 Quasispecies during Antiviral Treatment Dissected Using Ultra-Deep Pyrosequencing
Source: PLoS One. 2010 Jul 7;5(7):e11345. doi: 10.1371/journal.pone.0011345 (PMC2898805; doi:10.1371/journal.pone.0011345)
Supplement: Table S1 — Primers used for PCR amplification. (0.03 MB DOC) [file pone.0011345.s002.doc]

| **Table S1.** Primers used for PCR amplification | | | |
| --- | --- | --- | --- |
|  | **Direction** | **Primer sequence** | **Position in HxB2** |
| **Outer PCR** |  |  |  |
| JA 269 | Forward | AGGAAGGACACCARATGAARGA | 2045 - 2063 |
| JA272 | Reverse | GGATAAATCTGACTTGCCCART | 3343 - 3361 |
| **Inner PCR** |  |  |  |
| JA 329 | Forward | **GCCTCCCTCGCGCCATCAG***NNNN*GTAGCATGACAAAAATCTTAGAGCC^a^ | 3034 - 3058 |
| JA 331 | Reverse | **GCCTTGCCAGCCCGCTCAG***NNNN*TTCATAICCCATCCAAAGRAATG^a^ | 3248 - 3226 |

Footnotes

^a^ The UDPS adaptor regions are denoted in bold (JA 329 contains adaptor A and JA331 contains adaptor B), the sample-specific tag sequence region are italic and the gene specific primer regions are underlined.
