# Supplementary material for: Dynamics of HIV-1 Quasispecies during Antiviral Treatment Dissected Using Ultra-Deep Pyrosequencing
Source: PLoS One. 2010 Jul 7;5(7):e11345. doi: 10.1371/journal.pone.0011345 (PMC2898805; doi:10.1371/journal.pone.0011345)
Supplement: Table S2 — Tag sequences. (0.03 MB DOC) [file pone.0011345.s003.doc]

| **Table S2.** Tag sequences | |
| --- | --- |
| 1 | TACG |
| 2 | ACGT |
| 3 | CGTA |
| 4 | GTAC |
| 5 | TCAG |
| 6 | AGTC |
| 7 | CTGA |
| 8 | GACT |
