# Supplementary material for: Dynamics of HIV-1 Quasispecies during Antiviral Treatment Dissected Using Ultra-Deep Pyrosequencing
Source: PLoS One. 2010 Jul 7;5(7):e11345. doi: 10.1371/journal.pone.0011345 (PMC2898805; doi:10.1371/journal.pone.0011345)
Supplement: Table S3 — Genetic diversity of the 10 most common variants at each sampling time point. (0.03 MB DOC) [file pone.0011345.s004.doc]

| **Table S3.** Genetic diversity of the 10 most common variants at each sampling time point | | | | | | | | |
| --- | --- | --- | --- | --- | --- | --- | --- | --- |
| Patient | Time 1 | Time 2 | Time 3 | Time 4 | Time 5 | Time 6 | Time 7 | Time 8 |
| 1 | *1.41* | **2.29** | **2.20** | **1.65** | 1.40 | 1.28 | - | - |
| 2 | *1.99* | **1.36** | **0.87** | **1.10** | 2.18 | 2.06 | - | - |
| 3 | *2.61* | **3.02** | **0.94** | **0.91** | **0.93** | 5.77 | - | - |
| 4 | *1.79* | **2.98** | 2.55 | **1.62** | 2.02 | 2.02 | **2.19** | 1.91 |
| 5 | *1.60* | **2.19** | **2.67** | **1.60** | **1.62** | **1.93** | - | - |
| 6 | **3.11** | **3.94** | **4.16** | **2.58** | **1.89** | **1.64** | 1.68 | 0.99 |
| Footnotes  *^a^* Variants in italic are sampled before any treatment is initiated.  ^b^ Variants in bold are sampled during treatment failure.  ^c^ Variants in normal text are sampled during treatment interruption. | | | | | | | | |
